# The molecular epidemiology of SARS-CoV-2 in the Pityusic Islands shows multiple introductions and fast replacements of variants in a touristic worldwide hot spot

**DOI:** 10.1038/s41598-023-44668-5

**Published:** 2023-10-23

**Authors:** T. Viver, C. López-Causapé, P. Ribot-Fraile, C. Pérez-Mazón, D. López-Solé, G. Jiménez-Guerra, B. Taltavull, A. López-López

**Affiliations:** 1 Marine Microbiology Group, Mediterranean Institute for Advanced Studies (IMEDEA-CSIC-UIB), Esporles, Spain; 2 Servicio de Microbiología, Hospital Universitario Son Espases, Majorca, Illes Balears Spain; 3 Instituto de Investigación Sanitaria Illes Balears (IdISBa), Majorca, Illes Balears Spain; 4 Servicio de Microbiologíaa y Parasitología, Hospital Can Misses, C/ Corona s/n, 07800 Ibiza, Illes Balears Spain

**Keywords:** Epidemiology, Policy and public health in microbiology

## Abstract

The public health emergency caused by the Covid-19 outbreak in March 2020 encouraged worldwide initiatives to monitor the genetic diversity and features of the SARS-CoV-2 circulating variants, mainly based on the genomic surveillance. However, due to the impossibility to carry out extensive sequencing in resource-limited hospitals, other PCR-based strategies could be applied to efficiently monitor the circulating variants without the need to greatly expand the sequencing capacity. In our case, overpassing the technical limitations inherent to a second level hospital, we were able to characterize the weekly distribution of SARS-CoV-2 by the RT-qPCR amplification patterns visualization, single nucleotide polymorphism genotyping, and sequencing of randomly selected samples. All these molecular approaches allowed us to trace the epidemiology of SARS-CoV-2 viruses circulating in Ibiza and Formentera (Balearic Islands, Spain) during the third to the sixth pandemic waves (January 2021–July 2022), in which three major lineages that were considered as VOCs (Alpha, Delta, and Omicron), and many other non-VOC variants were detected and tracked.

## Introduction

The Pityusics are placed in the Western Mediterranean and comprise two major islands, Ibiza and Formentera, with a total population of about 160.000 habitants^1^. Both islands are major tourist destinations worldwide, with an important volume of domestic tourism and principal international connections to the UK and Germany^2^. However, the National lockdown imposed in Spain from 14 March to 4 May 2020 and the travel restrictions during years 2020 and 2021 to control COVID-19 dissemination increased the insularity of the area. These actions, together with the imposed social measures and the vaccination started at the beginning of 2021, affected the epidemiology of SARS-CoV-2 in our health area. The first analyses of the SARS-CoV-2 genomic epidemiology in the Balearic Islands were conducted with the samples taken during the three first pandemic waves, concluding that the Balearics constituted a unique setting in which the course of the pandemic was influenced by a complex interplay between insularity, severe social restrictions, and tourism travels^3^. In this work, we delved into the incidence and prevalence of the main SARS-CoV-2 circulating variants during a prolonged period of time (from the third to the sixth pandemic waves) in two of the main Balearic tourist destinations.

Since its detection in December 2019, the virus that causes severe acute respiratory syndrome or coronavirus disease COVID-19, has naturally accumulated mutations in its genome, leading to the emergence of multiple variants worldwide. Some of them have been classified as variants of concern (VOCs), because they have characteristics that imply the possibility of being more transmissible, causing a more serious disease, and even evading the natural immune and vaccination response^4^. The monitoring and follow-up of these variants have been fundamental to fight against the pandemic, and worldwide sequencing initiatives were advised as the best tool for the acute analyses of the mutations accumulated in the circulating variants and their spreading^5^. However, genomic tools, specifically genome sequencing technologies are not widely available, and even in developed countries the facilities needed for large-scale sequencing are limited^6^. This is the case of most county and regional, small hospitals in Spain, which depend on the reference hospitals sequencing services, and can only afford the analyses of a small representation of the total number of positives revealed by other molecular techniques.

Nevertheless, the visualization of the RT-qPCR amplification patterns as well as genotyping techniques can help significantly in delineating the epidemiology of SARS-CoV-2, providing rapid, cost-effective, and reliable ways to monitor SARS-CoV-2 variants circulating during the outbreak^5,7^.

Some specific PCR assays, by including the S gene among the analyzed viral targets, can identify variants carrying the Δ69–70 deletion, as the VOCs Alpha and Omicron BA.1 and BA.5^8^. Therefore, this target failure (Spike Gene Target Failure, SGTF) has been used as a screening test to track these VOCs, overcoming the necessity of sequencing. Moreover, other PCR assays (allele-specific RT-qPCR) were developed to identify the presence of other common mutations, in addition to the Δ69–70, emerging as potential solutions for identifying SARS-CoV-2 variants with potential therapeutics implications^9^. Viral genomes bearing the mutation of interest are selectively amplified in RT-qPCRs and then detected with a fluorescence-labeled oligonucleotide probe. These reactions can be multiplexed, allowing multiple mutations to be analyzed simultaneously^5^.

The identification of mutations associated with reduced vaccine efficacy or increased virus transmissibility has been of upmost importance in the delineation of effective strategies to mitigate and contain outbreaks of SARS-CoV-2 variants and other novel viruses^10^. Thus, we investigated the proportion of SGTF cases among the samples with a positive result for SARS-CoV-2 RT-qPCR as an approximation to know the frequency and permanence of the VOCs Alpha and Omicron BA.1/BA.5 in the Health Area of Ibiza and Formentera (ASEF), as well as single nucleotide polymorphism (SNP) genotyping to detect the VOCs Delta and Omicron BA.2 and BA.5. In addition, randomly selected positive samples were weekly collected for complete genome sequencing.

The main objective of this work was to show how the diversity, incidence, and prevalence of circulating SARS-CoV-2 variants were analyzed and evaluated in a second level hospital lacking the appropriate resources to carry out the epidemiological surveillance based solely on sequencing. By the followed strategy, we were able to obtain genetic information on the different circulating lineages of SARS-CoV-2 in our health area in the period covering from January 2021 to July 2022 (from the third to the sixth pandemic waves), contributing with valuable data to the COVID-19 Spanish Surveillance Consortium (https://www.seqcovid.csic.es), which ultimately has impacted the public health strategies of mitigation and containment of the virus in Spain.

## Results

### RT-qPCR detection of SARS-CoV-2 in the Health Area of Ibiza and Formentera

**Figure 1:**
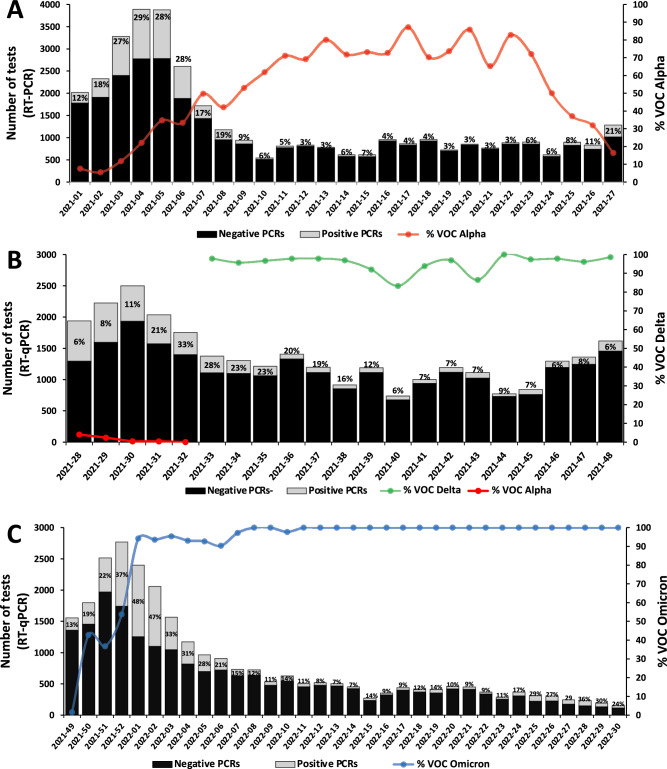
(**A**) Weekly number of analyzed samples, percentage of positivity (in red boxes) and frequency of the SGTF pattern (indicative of VOC Alpha detection) during the period covering from January to June 2021. (**B**) Weekly number of analyzed samples and percentage of positivity (in green boxes) during the period covering from July to November 2021. The frequency of detection of VOC Delta was not assessed by SGTF but by SNP genotyping. (**C**) Weekly number of analyzed samples, percentage of positivity (in blue boxes) and frequency of the SGTF pattern (indicative of VOCs Omicron BA.1 and BA.5) during the period covering from January to July 2022.

As shown in Table 1, 97,866 respiratory samples were processed for the diagnosis of COVID-19 during the study period (77,919 in 2021 and 19,947 in 2022). The number of SARS-CoV-2 positive samples was 16,946 in total, of which 12,073 were detected during 2021 and 4873 during 2022, representing a mean positivity of 12 and 15%, respectively. The highest rate of positivity in year 2021 took place during January (averaged 23%) and August (averaged 26%), corresponding to the 3rd and 4th–5th pandemic waves, with maximums of 29% and 33% in the fourth week of the year (week 2021–04) and the second of August (week 2021–32), respectively (Fig. 1A,B). In 2022, the peak in the positivity rate occurred during the 6th pandemic wave, with a historic maximum value of 48% in the second week of the year (week 2022–02, Fig. 1C). The mean positivity during the 6th pandemic wave was around 39%, which represents the maximum positivity record during a wave in our health area since the beginning of the Covid-19 pandemic.

**Table 1:** Monthly number of PCRs analyzed and mean positivity during the six pandemic periods included in the study.

Pandemic period	Year	Month	Total number of PCRs	Mean positivity (%)
3rd wave	2021	January	15,696	22,9
		February	7744	16,8
Inter-wave		March	4838	2,9
		April	3571	4,3
		May	3568	3,0
		June	3609	6,7
4th−5th waves		July	8311	25,9
		August	6844	19,9
Inter-wave		September	5194	7,3
		October	4481	6,6
		November	4785	8,1
6th wave		December	9278	21,2
	2022	January	8015	39,2
		February	3638	16,7
After-wave		March	2376	10,1
		April	1674	8,7
		May	1903	10,8
		June	1447	18,7
		July	894	29,5

### Incidence and prevalence of VOC Alpha by SGTF amplification pattern detection

The visualization of the RT-qPCR patterns obtained after the diagnostic procedure for the detection of SARS-CoV-2 allowed detecting the presence of the VOC Alpha (SGFT + pattern) in ASEF the last days of December 2020, coinciding with the onset of the third pandemic wave. From its first detection, both the mean global positivity and the frequency of the SGTF pattern showed a clear increasing trend, reaching at the end of January (week 2021–04) maximums of 28% and 35%, respectively (Fig. 1A). The maximum peak of detection of VOC Alpha was at the end of April 2021, when it represented up to 87% of the total number of the positives identified in ASEF by RT-qPCR. During February (week 2021–07), the positivity rate started to decrease until reaching values around 6% at the end of June (week 2021–24, Fig. 1), when the 4th and 5th pandemic waves started. At that time, the detection of the SGTF pattern fell to 4%, while the positivity rate increased up to 33%. The weekly number of analyzed samples, the percentage of positivity and the frequency of the SGTF pattern (indicative of Alpha variant detection) during this period are shown in Fig. 1A.

### Incidence and prevalence of VOC Delta by genotyping analyses

We could implement the mutation panel for VOC Delta genotyping in mid-August 2021, so during some period we only had evidence of the increasing proportion of the VOC Delta through the analyses of the sequencing data obtained from the randomly selected sequences. The sequencing results weekly obtained from SARS-CoV-2 positive samples (see below) revealed that this VOC emerged in ASEF in late May 2021 (week 2021–22), becoming rapidly the predominant one throughout the 4th and 5th pandemic waves. As shown in Table 2, the genotyping panel showed that most of the samples (96%) were affiliated to VOC Delta (the remaining 4% were not amplified, Cts > 32). In fact, until the end of the year, it was by far the predominant circulating variant in our health area (Fig. 1B). During this period, 1319 positive samples were genotyped, of which 57 were not amplified (Cts > 32) and 1246 were confidently affiliated to VOC Delta (Table 2). Thereafter, and coinciding with the 6th pandemic wave, we only could afford the genotyping of the positives obtained one day per week (385 in total). One hundred and thirteen samples were affiliated to VOC Omicron BA.1 by direct visualization of the amplification patterns (see below). The rest were genotyped (272 in total), of which 37 were not amplified (Cts > 32), and 235 were affiliated to VOC Delta, corresponding to 96% of the total positive samples (Table 2).

**Table 2:** Affiliation of the genotyped samples.

	Genotyped samples	VOC delta (%)	VOC omicron BA.2 (%)	NA (%)
**2021**				
August	438	426 (97.3%)	0	12 (2.7%)
September	251	242 (96.4%)	0	13 (5.2%)
October	259	213 (82.2%)	0	26 (10%)
November	371	365 (98.4%)	0	6 (1.6%)
December	552	399 (72.3%)	0	40 (7.2%)
**2022**				
January	51	37 (72.5%)	10 (19.6%)	4 (7.8%)
February	83	7 (8.4%)	70 (84.3%)	6 (7.2%)
March	180	1 (0.6%)	174 (96.7%)	5 (2.8%)
April	134	0	128 (95.5%)	6 (4.5%)
May	182	1 (0.5%)	168 (92.3%)	13 (7.1%)
June	121	4 (3.3%)	76 (62.8%)	41 (33.9%)

*NA* Not amplified.

From the second week of December (week 2021–50) to the end of the study period there was an almost complete displacement of the VOC Delta by the VOC Omicron, which became the predominant VOC throughout year 2022 (Fig. 1C).

### Incidence and prevalence of VOC Omicron subvariants by SGTF amplification pattern detection and genotyping

The visualization of the RT-qPCR patterns allowed detecting the presence of the VOC Omicron BA.1 (SGFT + pattern) in ASEF at the beginning of December 2021 (week 2021–49), when the mean positivity began to increase, and the sixth wave started in Spain. Both, the mean global positivity and the frequency of the SGTF + pattern showed an increasing tendency along January 2022, reaching values during the first week of the year of 48% and 94%, respectively (Fig. 1C). The genotyping panel used for the VOC Delta discrimination was also useful to confidently discriminate the VOC Omicron BA.2, the “stealth” Omicron, which emerged in our health area in January 2022. As shown in Table 2, 751 positive samples were genotyped, obtaining 50 samples affiliated to VOC Delta (7%) and 626 to VOC Omicron BA.2 (83%), whereas 75 samples (10% of the total) could not be amplified (Cts > 32).

Seven weeks after the onset of the 6th wave, the positivity rate declined, reaching a value of around 7% on early March (week 2022–13). However, the frequency detection of VOC Omicron did not change, remaining close to 100% during the rest of the analyzed period. In contrast, the proportions of the different subvariants within Omicron lineage were highly variable along the time, starting with a progressive replacement of BA.1 by BA.2, which became the dominant subvariant from February to May/June 2022 (see below). Subvariants BA.3 and BA.4 were detected in very low proportions along the year, being BA.5 the one that progressively replaced BA.2 in the last period (see below). The number of genotyped samples and the frequencies of VOCs Omicron and Delta from early December 2021 (week 2021–49) to July 2022 (week 2022–30) are detailed in Table 2.

### SARS-CoV-2 genetic diversity in ASEF as revealed by sequencing: Phylogenetic analyses

Whole genome sequences were obtained from 916 randomly selected among the positive respiratory samples. The accession codes of the sequences deposited in Gisaid (gisaid.org) can be found in Supplementary Table S1. Only 27 almost complete genome sequences, marked in bold in Supplementary Table S1, could not be deposited in Gisaid because did not fulfill the length requirements of this database. However, these sequences were used with confidence for the lineage assignment with the pangolin tool since all of them could be considered as high quality sequences with high coverage level (96% of the reference sequence). All genomes are publicly available at the repository of Discovery Environment (Cyverse) through the following link: https://data.cyverse.org/davanon/iplant/home/tomeu/Viver_et_al_2023/GENOMES_COVID.zip.

**Figure 2:**
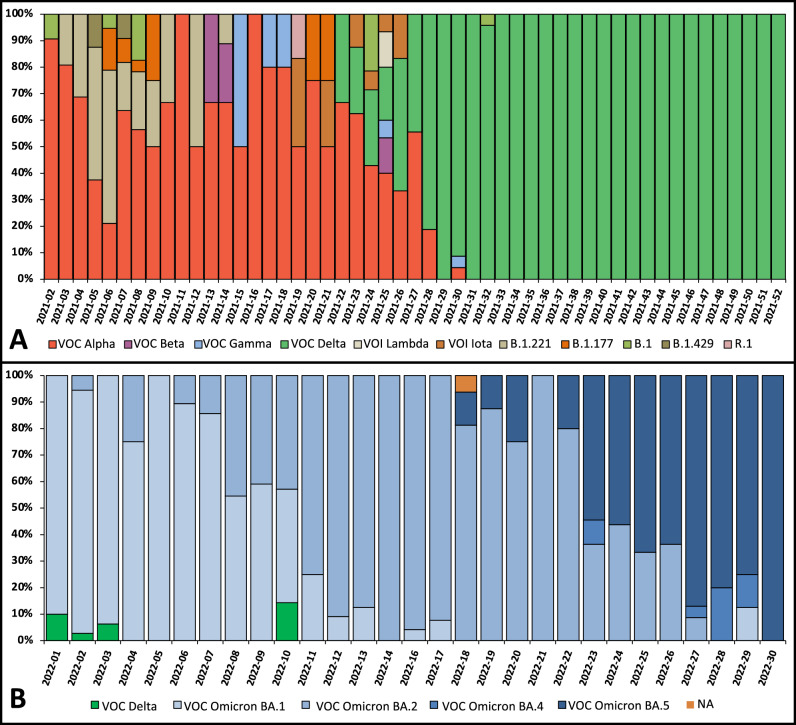
Sequencing results obtained from the randomly, weekly selected positive SARS-CoV-2 samples in years 2021 (**A**) and year 2022, from January to the end of July (**B**).

**Figure 3:**
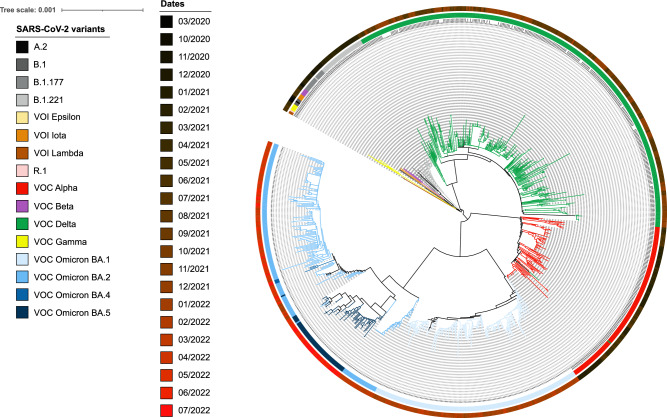
Phylogenetic reconstruction of a collection of 917 complete sequenced SARS-CoV-2 genomes. All SARS-CoV-2 variants have been represented in different colours in the internal ring, as indicated in the legend. In the external ring, grey gradient colours indicates the sampling dates (in months) for year 2020, gradient colour from black to brown for samples collected in 2021, and brown to red for samples obtained in 2022. Bar indicates 0.1% sequence divergence.

During year 2021, up to 543 of the generated consensus sequences (99.4%) were suitable for Pangolin lineage assignment. As shown in Fig. 2A, and concordantly with RT-qPCR amplification patterns analyses and genotyping, the VOCs Alpha and Delta were identified as the two major lineages circulating in ASEF in 2021, representing 32% and 50% of the total sequences, respectively. Lineage B.1.221 was detected in 10% of the analyzed samples, distantly followed by lineage B.1.177 (2%), VOC Gamma (1%), VOI Iota (1%), VOC Beta (0.8%), lineage B.1 (0,6%), VOI epsilon (0.6%), VOI lambda, and lineage B.1.165 (0.3% each), being VOI “Variant of Interest”. During the third epidemic wave (week 01–week 05 in 2021), the VOC Alpha and lineage B.1.221 were the most dominant variants, appearing at different proportions along this period (averaging 76% and 27%, respectively), although a very small number of other variants were also identified, including VOCs Beta and Gamma (Fig. 2A). The VOC Alpha remained the most prevalent variant until mid-June, when the 4–5th epidemic curve began in Spain and the VOC Delta emerged in our health area. The phylogenetic analyses (Fig. 3) also showed that VOC Delta gradually increased its proportion and displaced completely other variants by middle July (week 2021–28), in concordance with the fact that it was the only variant detected by sequencing and genotyping until the end of the year (Fig. 2A and Table 2). In year 2022, 373 sequences were suitable for Pangolin assessment, being those affiliated to VOC Omicron BA.1 the most detected at the beginning of the year (accounting for 82% of the total sequences obtained during the first three weeks, Fig. 2B). BA.1 was progressively replaced, first by BA.2 and finally by BA.5, that were the dominant subvariants from the middle March to June (week 2022–11 to week 2022–22), and at the end of the study period (from week 2022–23 to week 2022–30), respectively (Figs. 2B and 3).

## Discussion

SARS-CoV-2-related outbreak was declared by the World Health Organization (WHO) as a public health emergency of international concern on 30 January 2020, due to an increase in the number of imported cases worldwide^11^. First cases in Balearic Islands were detected by the end of March 2020, when initiatives to monitor the genetic diversity and features of the circulating variants, mainly based on the genomic surveillance, were established by the Spanish Health System^12^.

The first study addressing the evolution of SARS-CoV-2 infections in the Balearic Islands concluded that its relatively small size and the organization of health and epidemiological surveillance systems made the official accounts of reported cases more reliable than in other areas^2^. However, due to the impossibility to carry out extensive genomic sequencing in most of the islander hospital facilities, the monitoring and tracking of the circulating variants had to rely in other PCR-based strategies, such as the detection of the SGTF genomic signature and/or high-throughput genotyping methods.

The SGTF genomic signature for the detection of the Alpha and Omicron variants (B.1.1.529, BA.1, BA.4 and BA.5) provide a very rapid and accurate proxy for their identification. In fact, at the peak of their corresponding wave, the positive predictive value of the SGTF was 98% for Alpha and 100% for Omicron^13^. On the other hand, the genotyping RT-qPCR methods have showed increased sensitivity over sequence-based approaches, allowing for their quantitation, and providing readily interpretable results within hours. However, designing assays for VOCs is a process requiring continuous adaptation and validation since emerging VOCs may possess unique mutations that necessitate the development of new assays^5^. Specifically, TaqMan SARS-CoV-2 mutation panel molecular genotyping assays allow the detection and differentiation of the most common variants that have been used for surveillance and epidemic control and prevention: B.1.1.529 (Omicron), B.1.617.2 (Delta), B.1.1.7 (Alpha), B.1.526 (Iota), B.1.351 (Beta), P.1 (Gamma), P.2 (Zeta), B.1.617.1 (Kappa), and B.1.427/B.1.429 (Epsilon)^14^.

These strategies allowed us to detect the predominant lineages and VOCs in each pandemic period, as well as the emergence and extinction of the different lineages through time. Specifically, the dynamics of the SARS-CoV-2 variants in our health area was characterized mainly by the amplification patterns visualization and SNP genotyping, whereas sequencing was only performed in weekly, randomly selected samples.

Overall, the most prevalent variant during the first half of year 2021 was the VOC Alpha, which was first detected in ASEF on the 29th of December 2020 and strongly associated with an increase in the incidence. Interestingly, during this period other lineages carrying multiple mutations in the spike and considered as VOCs by the WHO and the European Center for Disease Control (ECDC) emerged in our area, namely the VOCs Beta and Gamma, although their prevalence remained low. The replacement of these VOCs and other non-VOC lineages occurred progressively, and VOC Alpha did not become majoritarian until March 2021, when it accounted for more than 50% of the total number of positives detected. Of note, the VOC Alpha did not trigger a high hospital burden in Spain nor in Balearics, but it likely contributed to high community transmission rates due to its enhanced transmissibility and the increasing social interactions during that period. The restrictive measures established in the Pityusics during 2020 to control the movement of travelers in ports and airports were progressively relaxed during 2021, restoring in part the social interactions, the family and mass gatherings, and the possibility of traveling to and from the islands.

During the second half of year 2021, when the vaccination campaign was in an advanced stage, with about 80% of the ASEF population immunized, the VOC Delta was by far the predominant one (see Table 2 and Fig. 2A). From its first detection in late May 2021, it rapidly increased in prevalence, out-competing pre-existing lineages and becoming the most common variant (almost the totality of the cases detected from July to late November), as occurred in many other Spanish locations and in other countries^15,16^. The emerging of VOC Delta was associated with a rapid surge of COVID-19 cases in the area, provoking the fourth and fifth waves and the highest number of positive cases detected during 2021. It is now known that the competitive advantage of the Delta variant relied in two main characteristics, its increased transmissibility, with higher infectious virus loads, and its resistance to natural and vaccine-induced immunity, which probably contributed to the rapid and intense transmission in the Pytiusics^17,18^. However, nationwide records of COVID-19 hospitalizations in Spain indicated that the mass vaccination prevented a fourth wave of hospitalization after Eastern holidays^19^.

The VOC Omicron was detected in December 2021, becoming dominant across the area in less than one month, significantly increasing SARS-CoV-2 transmission, even when the vaccinated population was around 90%. This VOC accumulated many mutations that conferred a marked advantage over pre-circulating lineages, causing a major upsurge of cases in multiple countries worldwide since November 2021^20^. The selective advantage of Omicron over Delta may be explained again by increased transmissibility and by higher immune escape^21^.

The introduction of vaccines at the end of 2020, prioritizing the elderly and healthcare workers, and the subsequent steady vaccination by age groups, could have had an effect in favoring the selection of vaccine-scape mutants, although no evidence has been found in favor of this threat. On the contrary, a number of published studies uniformly highlight that changes in neutralization activity were not translated into complete vaccine failure with symptomatic illness^22,23^. In fact, vaccine protection remains clinically evident based on the decrease in the rate of COVID-19 hospitalizations in Spain since vaccination started^19^.

Summarizing, the SARS-CoV-2 epidemics in ASEF was characterized by four differentiated phases: (i) in a first stage, a progressive replacement of non-VOC variants by VOC Alpha during the first half of year 2021 and a total replacement of VOC Alpha by VOC Delta during the second half was observed; (ii) in a second stage, a fast replacement of VOC Delta by VOC Omicron BA.1 during December 2021 and early January 2022 was detected, (iii) during the third one, it was detected a rapid replacement of Omicron BA.1 by Omicron BA.2 (from March to June 2022), and lastly, a fast replacement of BA.2 by BA.5, from mid-2022 to the end of the studied period. As has been extensively reported, the VOC Omicron did not suppose a public health concern in our area, since has reduced probability of hospital admission, with shorter period of illness and less infectiousness potential^24^.

The relative geographical isolation of the Balearic Islands and their central role as a hub for international tourism along with the extension of vaccination, affected the COVID-19 pandemic in the Health Area of Ibiza and Formentera^3^, outlining the introduction, spread and extinction of different lineages and VOCs through time. The comparative study of the epidemiology of SARS-CoV-2 in different Spanish regions across the six epidemic waves reported that leading European tourist destinations, as Balearics, could explain the high diversity of circulating variants observed as compared with others worldwide^25^.

As SARS-CoV-2 became endemic in most areas, local governments relaxed the quarantine policies and public health measures for a gradual return to normal life. Although the number of confirmed cases was gradually decreasing during years 2022 and 2023, novel variants of SARS-CoV-2 could continuously threaten public health^26^. The multiple introductions and fast replacement of VOCs detected in this work reinforce the idea that the variant monitoring is an effective strategy for the virus control, in addition to other initiatives such as vaccination, social measures, hygiene, and health education. Finally, it is important to emphasize that after having gone through a pandemic with serious repercussions on the health at a global scale, it is more necessary than ever before increasing research funding and encouraging international collaborations as possible potential solutions for the sustainable future improvement of genomic epidemiology research in developing countries^6^.

## Methods

### Sample collection

For SARS-CoV-2 diagnosis, nasopharyngeal samples were collected in 3 ml vials with minitip flocked swabs, designed for the conservation and transport of respiratory pathogens (“KaiBiLi” viral transport medium, Genesis). The vials were transported at 4 °C from the different sampling centers to the Can Misses Hospital, individually bagged in order to avoid possible cross contaminations.

We analyzed the daily diagnostic data of SARS-CoV-2 obtained by RT-qPCR in the Molecular Biology Laboratory of the Microbiology and Parasitology Service of the Can Misses Hospital, in the period covering from 1st January 2021 to 31st July 2022. Our laboratory received samples from all public health centers and services belonging to ASEF, comprising two hospitals (located in Ibiza and Formentera), 59 health centers, and 104 primary care units, scattered over the two islands, belonging to the Balearic Public Health System (Ib-Salut) and the Spanish Government Health System (SNS).

One day per week, randomly selected positive samples (1040 in total) were sent to the Microbiology Department of Son Espases University Hospital) for sequencing, where they were stored frozen at -80ºC until use.

### Nucleic acid extractions and amplification of viral genes

After vigorous vortexing of the vials, 200 µl of homogenized samples were used for extractions. To obtain the genetic material, extraction was performed with MagMax™-96 Viral RNA Isolation Kit (AM1836, Applied Biosystems) and the KingFisher automatic extractor (Thermofisher), following the protocols indicated by the manufacturers. Once the process was finished, the viral purified nucleic acids were eluted in 53 µl of the elution buffer provided in the kit.

Subsequently, the amplification of three viral gene fragments was performed with 10 µl of the extracted nucleic acids with the "TaqPath™ COVID-19 CE-IVD RT-PCR" diagnostic kit from Applied Biosystems (A48067, Thermofisher) in a QuantStudio 5 thermocycler (Thermofisher), as recommended by the manufacturer. The RT-PCR assay is aimed at the amplification of three specific regions of the SARS-CoV-2 genome contained in the ORF1ab, N and S genes. The thermal protocol consists in 40 amplification cycles (3 s at 95 °C, 30 s 60 °C), preceded by 2 min at 25 °C (UNG incubation) and 10 min at 53 °C (retrotranscription). Data were analyzed with the QuantStudio Design and Analysis Software v2.4 and the COVID-19 Interpretive Software (Thermofisher).

### Detection of VOCs Alpha and Omicron BA.1/BA.5 by amplification patterns visualization

Depending on the amplification pattern obtained after diagnostic RT-qPCR, positive samples were classified into two groups: those with the failure in the amplification of the S gene fragment (SFGT + samples, corresponding to VOCs Alpha and Omicron BA.1/BA.5, depending of the period of time), and those with the typical amplification pattern in which the S gene is normally amplified (SFGT-, corresponding to other variants). We could discern accurately between the different VOCs with SFGT + pattern because they did not coexist in our health area. Specifically, the Alpha variant was no longer circulating in July 2021 whereas VOC Omicron appeared in December 2021. The same rationale was applied to distinguish between Omicron BA.1 and BA.5, since the first became extinct in our health area when the second started to be detected.

### Detection of VOCs Delta and Omicron BA.2 by genotyping analyses

In the positive samples with the typical amplification pattern (SGFT-), we could detect the Delta and Omicron BA.2 variants by using the Applied Biosystems TaqMan SARS-CoV-2 Mutation Panel assay L452R (CVAAAAD, Thermofisher) combined with TaqPath 1-Step RT-qPCR Master Mix (A15300, Thermofisher), following the manufacturers´ instructions. Genotyping was performed with a QuantStudio5 Real-Time PCR Instrument in all the positive samples identified by RT-qPCR with Ct values ≤ 32 (Ct = 30 is the maximum Ct value defined in the manufacturer´s protocol, although we were able to unequivocally genotype samples with Cts ≤ 32). Data were analyzed with the QuantStudio Design and Analysis Software v2.4 with the Genotyping Analysis Module. Based on the allelic discrimination plot, the reference allele that clusters along the X-axis (allele 1 VIC dye) is represented as homozygous allele1, and the mutant allele that clusters along the Y-axis (allele 2 FAM dye) is represented as homozygous allele 2. These cluster plots show very clear discrimination between the wild-type samples and the mutation samples^8^. During the 6th pandemic wave, from December 2021 to late January 2022, we could only afford the genotyping of the positives obtained one day per week, as the positivity rate increases considerably, and the high quantity of samples were not manageable in our facilities. During these two months, the positive samples obtained in days 9, 13, 21, and 28 of December 2021 (representing weeks 48–52 2021) and days 4, 11, 18 and 25 of January 2022 (weeks 1–4 2022) were selected for genotyping. Thereafter, and comprising the period from February to the end of June 2022, again all the positive samples with SFGT- pattern obtained were genotyped.

### Genomic library preparation and sequencing

Genomic library preparation was conducted following the SeqCovid Consortium protocols^11^. Briefly, RNA was first retro-transcribed into cDNA and SARS-CoV-2 complete genome amplification was conducted in two parallel multiplex PCR, accordingly to the openly available protocol developed by the ARTIC network^27^ and using the V3, V4 y V4.1 multiplex primers scheme. Resulting amplicon pools were then combined and cleaned using AMPure beads (Beckman Coulter), and 50 ng were used to prepare the Illumina sequencing libraries (Illumina DNA Prep kit, Illumina Inc., San Diego, CA) according to the manufacturer’s protocol and with 5 cycles for indexing PCR (Nextera DNA CD Indexes, Illumina). Finally, indexed genomic libraries were pooled in equimolar amounts and loaded onto a MiSeq v3 cartridge (2 × 250 cycles).

### Lineage assignment and spike mutation surveillance

Two different bioinformatic approaches were used for whole genome sequence analysis: an open source pipeline based on IVAR (https://gitlab.com/fisabio-ngs/sars-cov2-mapping) and the DRAGEN COVID Lineage App available version (Illumina®). Pipelines, map quality- and primer-trimmed viral reads to the hCoV-19/Wuhan/WIV04/2019 reference sequence genome (MN908947.3/NC_045512.2), result in the generation of consensus whole genome sequences.

Consensus sequences covering at least 75% of the reference sequence and with a median coverage > 100 reads were considered for lineage assignment. For this purpose, the Phylogenetic Assignment of Named Global Outbreak Lineages (PANGOLIN) tool was used (https://github.com/cov-lineages/pangolin), employing the latest version and the most updated lineage database available at the time of assignment. Detection of additional spike amino acid substitutions was conducted by using the Basic Local Alignment Search Tool (BLAST) and the spike protein of the hCoV-19/Wuhan/WIV04/2019 strain as reference.

### Phylogenetic analyses

The 916 complete sequenced SARS-CoV-2 genomes were aligned using the MUSCLE tool v3.8.31^28^. The phylogenetic analyses and tree reconstruction were carried out using the neighbour-joining algorithm implemented in the ARB software (v6.0.6) using the Jukes-Cantor correction^29^. The obtained phylogenetic tree was annotated and edited using the on-line tool “Interactive Tree of Life (iTol)”, available at https://itol.embl.de^30^.

### Ethics statement

The results presented in this study are derived from routine clinical practice, so it was not necessary to obtain informed consent from patients as not clinical data has been used and patients did not undergone any additional procedure. Son Espases University Hospital is the designated reference laboratory for the Balearic Islands in the Spanish network for genomic surveillance of SARS-CoV-2 (RELECOV)^31^.

### Supplementary Information

Supplementary Table 1.

## Data Availability

The datasets obtained in this study can be found in Gisaid database (https://gisaid.org), under the accession codes listed in Supplementary Table S1 and in the link: https://data.cyverse.org/davanon/iplant/home/tomeu/Viveretal.2023/GENOMESCOVID.zip.
